# Diol responsive viscosity increase in a cetyltrimethylammonium bromide/sodium salicylate/3-fluorophenylboronic acid micelle system

**DOI:** 10.1039/d1ra08831a

**Published:** 2022-02-25

**Authors:** Ryotaro Miki, Tsutomu Yamaki, Masaki Uchida, Hideshi Natsume

**Affiliations:** Faculty of Pharmacy and Pharmaceutical Sciences, Josai University 1-1 Keyakidai, Sakado Saitama 350-0295 Japan rmiki@josai.ac.jp +81-49-271-7052 +81-49-271-7052

## Abstract

We report a novel smart micellar system utilising a phenylboronic acid (PBA) derivative whose viscosity increases on adding diol compounds such as sugar or sugar alcohol. We prepared a typical worm-like micelle (WLM) system in 100 mM cetyltrimethylammonium bromide (CTAB)/70 mM sodium salicylate (NaSal), which showed high zero-shear viscosity (*η*_0_). Upon the addition of 20 mM 3-fluorophenylboronic acid (3FPBA) to the WLM system, *η*_0_ decreased by 1/300 that of the system without 3FPBA. Furthermore, upon the addition of 1.12 M fructose (Fru) and 1.12 M sorbitol (Sor) to the CTAB/NaSal/3FPBA system, *η*_0_ increased by 50-fold and 30-fold, respectively. ^19^F NMR spectral results of the systems using 4-fluorosalicylic acid (FSal) instead of NaSal demonstrated that the FSal/3FPBA-complex interacts with CTAB. Moreover, the addition of sugar or sugar alcohol to the micellar system leads to a decrease in the amount of FSal/3FPBA-complex interacting with CTA^+^ and an increase in the amount of 3FPBA/Fru or Sor-complex, which does not interact with CTA^+^. These changes in molecular interactions induce the elongation of the WLMs and increase the viscosity of the system. This system utilises the competitive cyclic ester bond between the NaSal/3FPBA and 3FPBA/sugar or sugar alcohol to induce viscosity changes.

## Abstract 2

We report a novel smart micellar system utilising a phenylboronic acid (PBA) derivative whose viscosity increases on adding diol compounds such as sugar or sugar alcohol.

## Graphical abstract

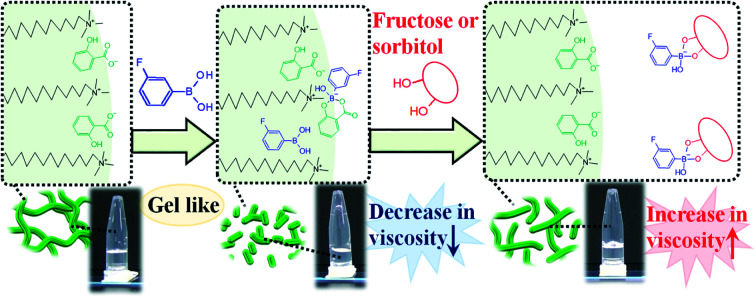

## Introduction

**Fig. 1:**
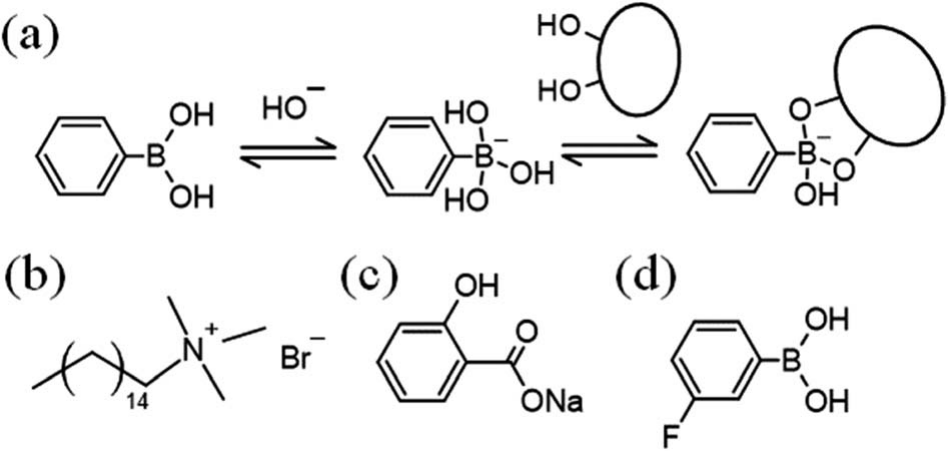
(a) Acid–base equilibrium of phenylboronic acid, and the binding equilibrium between PBA and *cis*-diol compounds. Chemical structures of (b) cetyltrimethylammonium bromide, (c) sodium salicylate, and (d) 3-fluorophenylboronic acid.

Worm-like micelles (WLMs) are molecular assemblies which are mainly formed by surfactants, and they show unique viscoelastic properties.^1,2^ Stimuli-responsive WLMs are expected to be smart materials with applications in a wide range of areas. A variety of stimuli-responsive WLMs, with stimuli such as light,^3–6^ redox reactions,^7,8^ pH changes,^9–11^ and CO_2_ ^12^ have been reported.^13^ Phenylboronic acid (PBA) is a Lewis acid and a functional molecule, since it reversibly forms cyclic ester bonds with *cis*-diol compounds such as sugars (Fig. 1a).^14^

This equilibrium shifts to react with diol compounds, leading to a decrease in the molecular form of PBA and an increase in the anionic form. Utilising this change, PBA has been studied as a sugar sensor for analytical chemistry and supramolecular assemblies.^15–17^ Sugar-responsive viscoelastic systems^18–21^ are desired for applications in fields such as drug delivery, analytical chemistry, cell engineering or medical engineering. Recently, novel sugar-responsive WLMs composed of a PBA or PBA derivative and a cationic surfactant, cetyltrimethylammonium bromide (CTAB, Fig. 1b) were reported, whose viscosity is decreased on adding diols such as sugars.^22,23^ However, to the best of our knowledge, there was no diol-responsive micellar system whose viscosity increases with the addition of diols. Herein, we report a novel diol-responsive viscosity-increasing micellar system.

## Experimental

### Materials

Sodium salicylate (NaSal, Fig. 1c), fructose (Fru), glucose (Glc), sorbitol (Sor), sodium hydroxide solution (1, 8 mol L^−1^), CTAB, sodium dihydrogen phosphate, disodium hydrogen phosphate, alizarin red S (ARS), and trifluoroacetic acid were obtained from FUJIFILM Wako Pure Chemical Co., Osaka, Japan. 3-Fluorophenylboronic acid (3FPBA, Fig. 1d) and 4-fluorosalicylic acid (FSal) were obtained from Tokyo Chemical Industry, Tokyo, Japan. Sodium deuteroxide (NaOD) solution (40% (w/w)) was purchased from Sigma-Aldrich, Tokyo, Japan. Deuterium oxide (D_2_O) was acquired from Kanto Chemical Co., Inc., Tokyo, Japan.

### Preparation of the mixed micellar systems

We prepared a mixed micellar system by mixing the two stock solutions under a few conditions. Stock solution A contained 200 mM CTAB or 200 mM CTAB with different concentrations of 3FPBA in distilled water. Stock solution B contained 140 mM NaSal and 200 mM phosphate, and its pH was adjusted to 7.4 using aqueous NaOH solution. Sugar or sugar alcohol was added as powder to System. The prepared mixed micellar systems were stored at 25 °C for more than 24 h after mixing at a temperature in the range of 15–25 °C or with heating using a hot magnetic stirrer.

### Observation of appearance

We prepared solutions (1.0 mL) in 1.5 mL microtubes or solutions (2.0 mL) in 6 mL glass vials. For microtubes, images were captured 3 s after the microtubes were inverted.

### Rheological measurements

A stress-controlled rotational rheometer (MCR-102, Anton Paar, Ostfildern, Germany) was used for steady and dynamic rheological measurements at 25 °C. We used a cone plate or concentric cylinder for both measurements. The strain (*γ*) was fixed at 10% for the dynamic viscoelasticity measurements.

### Particle size measurements

The mean particle size was measured by dynamic light scattering (DLS) at 25 °C with a Malvern Zetasizer Pro system (Malvern Panalytical, London, UK) equipped with a 4 mW and 633 nm He–Ne laser.

### Fluorescence measurements for determination of apparent binding constant (*K*)

Fluorescence spectra were recorded using a Shimadzu RF-5300PC instrument (Shimadzu Corporation, Kyoto, Japan). The *K* between 3FPBA and ARS, and *K* between 3FPBA and diol compounds was obtained according to a previously reported method.^14,24,25^

### NMR spectroscopy

^1^H and ^19^F NMR spectroscopy was conducted using a Varian 400 MR spectrometer (Agilent Technologies, CA, USA). For ^19^F NMR spectroscopy, trifluoroacetic acid was used as the external standard (chemical shifts (*δ*): −76.5 ppm), and the solvent used contained 10% D_2_O solution. For ^1^H NMR spectroscopy, the pD values were calculated on the basis of the apparent pH values measured in D_2_O using the following equation:^26,27^

1 pD = apparent pH + 0.44

The pD values were then adjusted using NaOD solution.

## Results and discussion

**Fig. 2:**
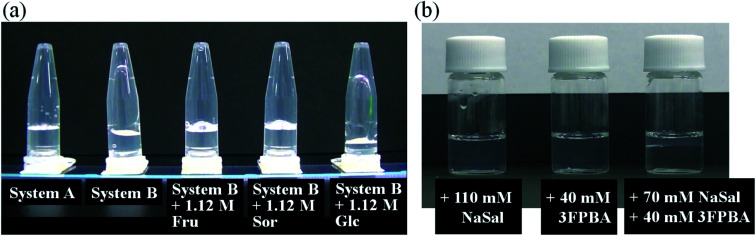
Visual appearance of (a) System A, System B, and System B with different diols, 3 s after the microtubes were inverted, and (b) 100 mM CTAB samples in 100 mM phosphate (pH 7.4) with 110 mM NaSal, 40 mM 3FPBA, and 70 mM NaSal + 40 mM 3FPBA.

It is known that boric acid forms cyclic ester bonds with salicylic acid^28^ and CTAB forms typical WLMs upon the addition of NaSal.^1,2^ Based on these reports, we hypothesised that adding a PBA derivative to the CTAB/NaSal WLM system induces a viscosity change. First, we prepared System A (pH 7.5) using 100 mM CTAB/70 mM sodium salicylate/100 mM phosphate, which is based on a typical WLM system,^1,2^ and System A with 3FPBA. System B was then defined as System A with 20 mM 3FPBA. When microtubes containing the systems were inverted, System A maintained a gel-like appearance, while System B dropped rapidly (Fig. 2a). We also prepared System B with sugars, Fru and Glc, and sugar alcohol, Sor as diol compounds. The sample with 1.12 M Fru or 1.12 M Sor dropped slowly compared to System B (Fig. 2a). The sample with 1.12 M Glc dropped as rapidly as in System B. To study the effect of 3FPBA addition to the phase state of the CTAB/NaSal system, we prepared a 100 mM CTAB/110 mM NaSal/100 mM phosphate system (System C), a 100 mM CTAB/40 mM 3FPBA/100 mM phosphate system (System D), and a 100 mM CTAB/70 mM NaSal/40 mM 3FPBA/100 mM phosphate system (System E). Systems C and D were transparent and existed as a single phase, whereas System E resulted in phase separation (Fig. 2b).

**Fig. 3:**
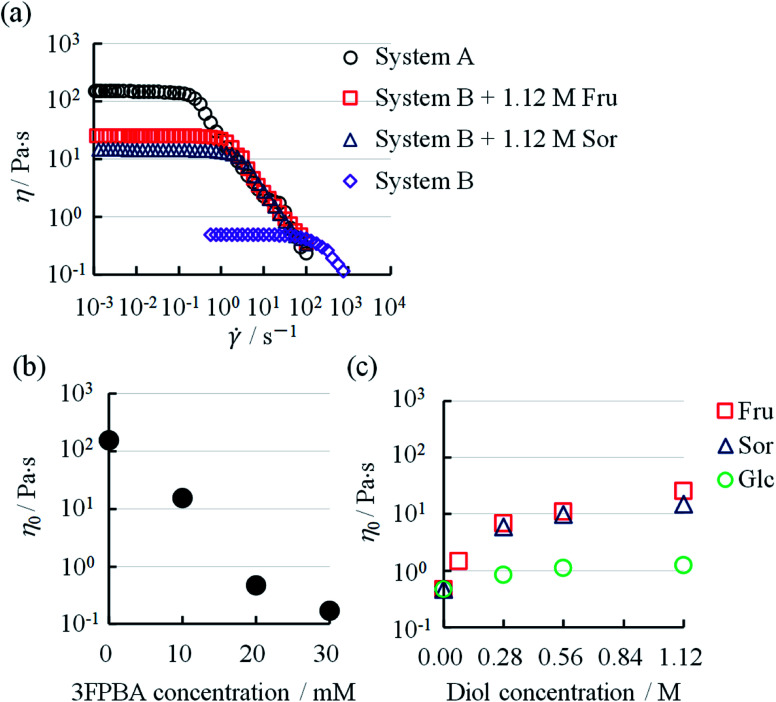
(a) Steady shear rate (*γ̇*) dependent viscosity (*η*) behaviours in System A, System B and System B with diols, (b) relationship between *η*_0_ and 3FPBA concentration in System A. (c) Relationship between *η*_0_ and diol concentration in System B.

To further investigate this unique phenomenon, we evaluated the rheological characteristics and the relationship between the shear rate (*γ̇*) and viscosity (*η*). In System A, *η* was a constant at low *γ̇* and decreased after a certain *γ̇* (Fig. 3a). Such a characteristic rheological property is observed in typical WLMs.^29,30^ Though System B and System B with 1.12 M Fru or 1.12 M Sor had different *η* at low *γ̇*, they showed similar rheological behaviours to that of System A. We obtained the zero-shear viscosity (*η*_0_) by the extrapolation of *η*, which is independent of *γ̇* at low *γ̇*, onto the *y*-axis. The *η*_0_ of System A decreased from 156 Pa s with increasing 3FPBA concentration, to 0.47 Pa s (1/300 compared to without 3FPBA) at 20 mM 3FPBA, and to 0.17 Pa s (1/900 compared to without 3FPBA) at 30 mM 3FPBA (Fig. 3b).

3FPBA was effective in decreasing the viscosity of System A. By contrast, the *η*_0_ of System B with sugar or sugar alcohol increased from 0.47 Pa s with increasing diol concentration (Fig. 3c). The *η*_0_ increased approximately 50-fold (25.5 Pa s) with 1.12 M Fru, 30-fold (15.0 Pa s) with 1.12 M Sor and 2-fold (1.23 Pa s) with 1.12 M Glc. Fru and Sor were more effective compared to Glc in increasing the viscosity of System B. The diols in decreasing order of effect on *η*_0_ is Fru > Sor > Glc.

To obtain further rheological data, we measured dynamic viscoelasticity. The dynamic viscoelasticity measurements revealed the behaviours of both the storage modulus (*G*′) and loss modulus (*G*′′) at different frequencies (*ω*). These parameters are based on the Maxwell model in eqn (2) and (3), respectively:2
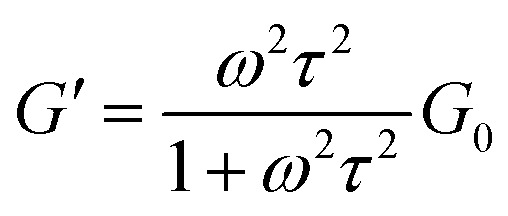
3
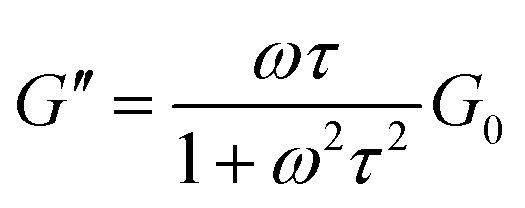
where *τ* and *G*_0_ are the relaxation time and plateau modulus, respectively. When the rheological behaviours follow the Maxwell model with a single *τ*, *G*′, and *G*′′ can produce a semicircular curve in the Cole–Cole plot (*G*′ *versus G*′′), as shown in eqn (4).^1,31^4
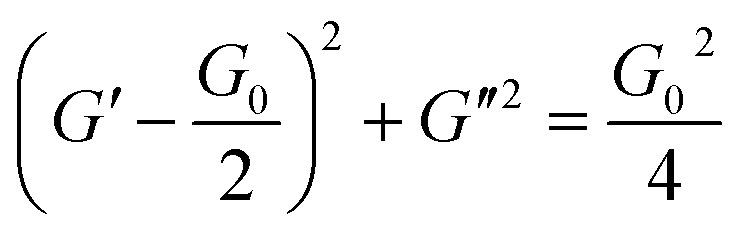


**Fig. 4:**
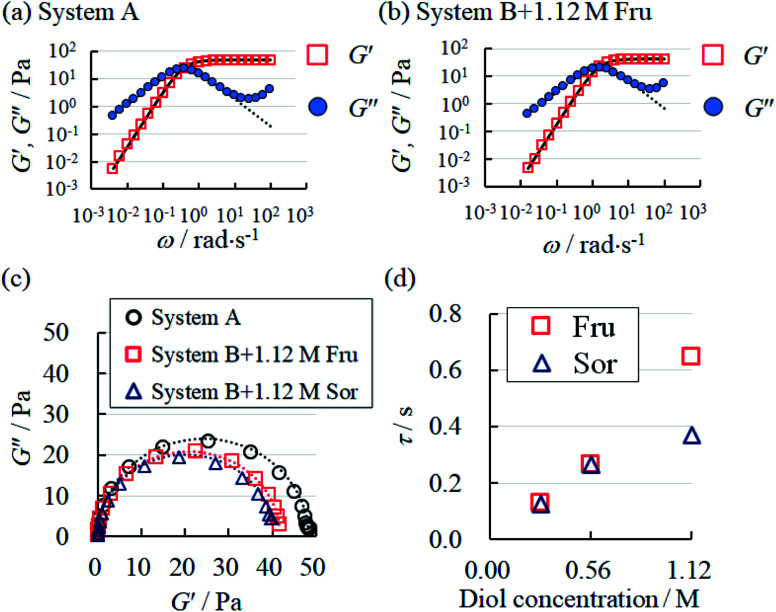
Frequency-dependent behaviours of storage modulus (*G*′) and loss modulus (*G*′′) in (a) System A, (b) System B with 1.12 M Fru. The dotted and solid curve fittings were calculated according to eqn (2) and (3), respectively. (c) The Cole–Cole plot of System A and System B with fructose (Fru) or sorbitol (Sor). The dotted lines represent the fitting curves obtained using eqn (4). (d) The behaviour of relaxation time (*τ*) in System B with Fru or Sor.

In both System A and System B with 1.12 M Fru, *G*′′ was higher than *G*′ at low *ω*, but lower at high *ω* (Fig. 4a and b). Neither System B nor System B with 1.12 M Glc had sufficient viscoelasticity for dynamic viscoelasticity measurements (data not shown). To study the formation of WLMs, we used a Cole–Cole plot, in which a semicircular curve is a rheological characteristic of typical WLMs.^1^ System A and System B with 1.12 M Fru or 1.12 M Sor showed near-perfect semicircular curves (Fig. 4c). Based on these rheological characteristics, we presumed that these samples formed adequately long and entangled WLMs. We analysed the change in entanglement of WLMs in System B with the addition of sugar or sugar alcohol using *τ*, an index of entanglement of WLMs.^29,32^ We defined *ω*_c_ as the intersection point of *G*′ and *G*′′ in the dynamic viscoelasticity measurements and obtained *τ* as the inverse of *ω*_c_. *τ* increased with increasing concentrations of Fru or Sor in System B (Fig. 4d). The *τ* of System A was 2.6 s, which is larger than that of System B with 1.12 M Fru (0.65 s). From these results, we deduce that adding 3FPBA to System A induces shortening of WLMs and adding Fru or Sor reverses this change.

**Fig. 5:**
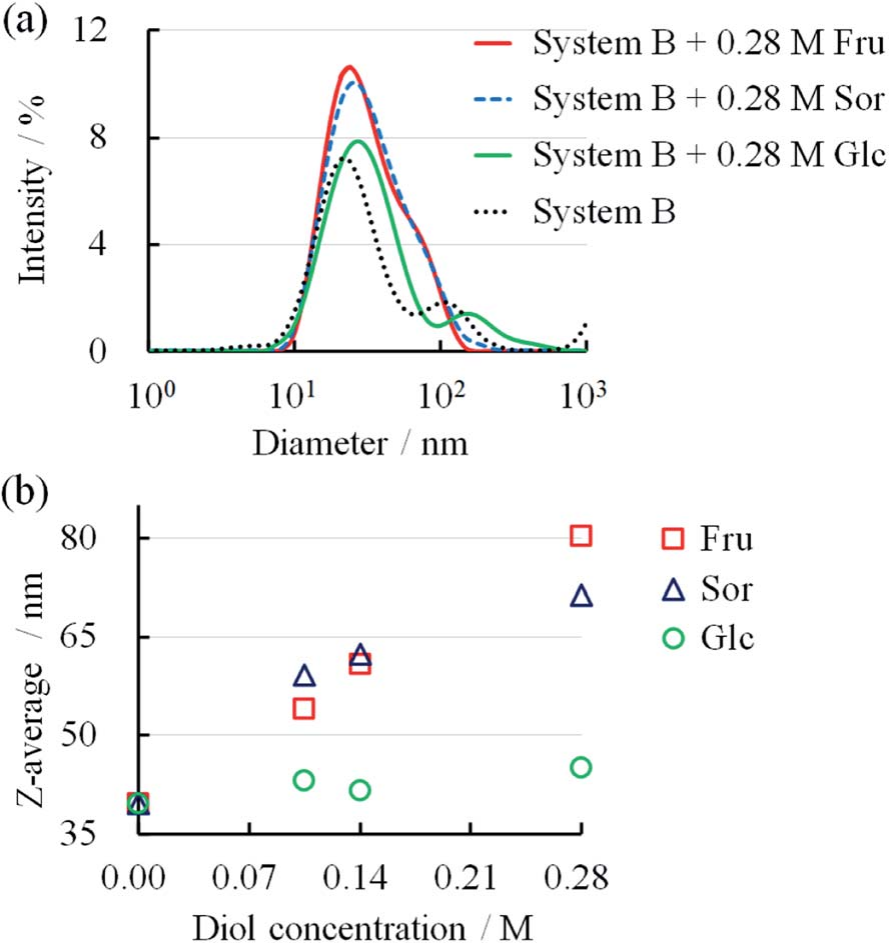
(a) Size distributions of System B with different diol concentrations. (b) Relationship between diol concentration and *Z*-average in System B.

DLS has been used to confirm the changes in the micellar forms.^12,22,33–35^ We determined the particle size through DLS to confirm the changes in the micellar systems upon the addition of diol compounds. The size distributions of System B and System B with diol compounds are shown in Fig. 5a. After the addition of 0.28 M Fru, Sor, and Glc, the peaks of the DLS spectra broadened, indicating larger size distributions. The mean particle sizes, in terms of *Z*-average, increased with an increase in diol compounds (Fig. 5b). Fru and Sor substantially affected the *Z*-average of the micellar systems in relation to Glc. These results showed that the micelles elongated upon the addition of diol compounds, particularly Fru and Sor. These results are consistent with those from the rheology measurements (Fig. 3c).

**Fig. 6:**
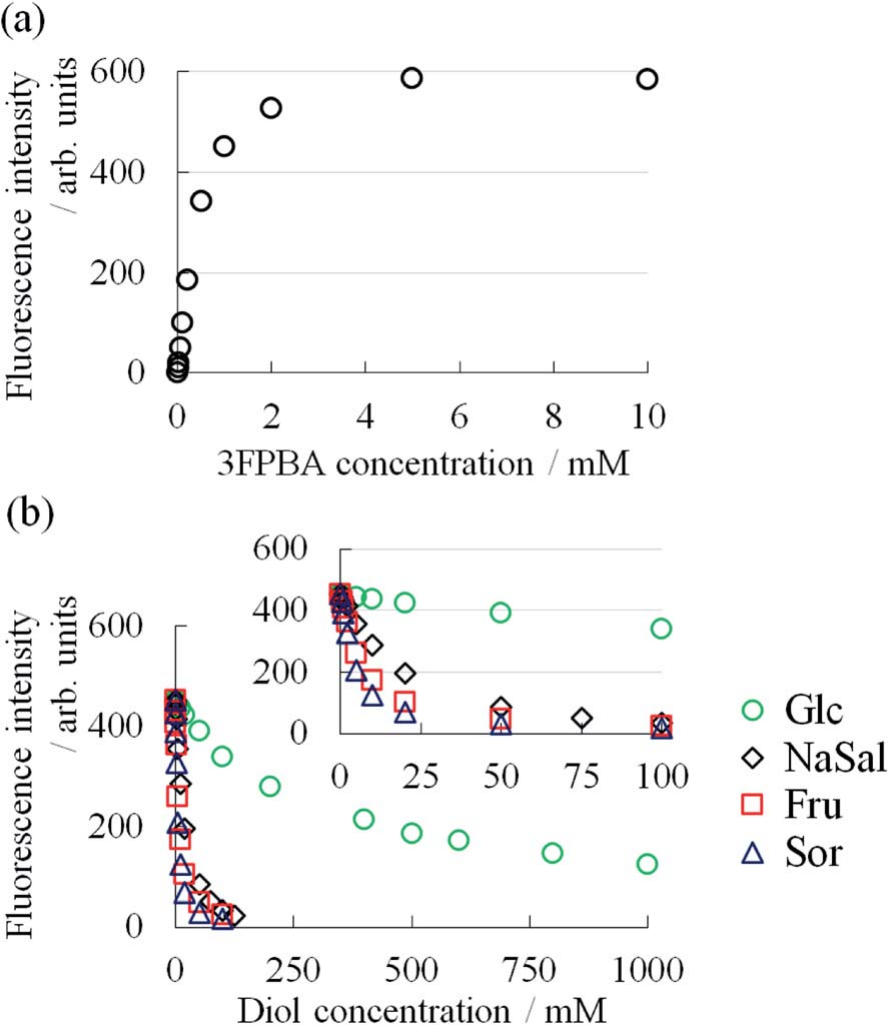
(a) Relationship between 3FPBA concentration and fluorescence intensity of ARS (0.10 mM) at pH 7.4 in 100 mM phosphate buffer (*λ*_ex_ = 495 nm, *λ*_em_ = 565 nm). (b) Relationship between diol compounds concentration and fluorescence intensity of ARS (0.10 mM) in the presence of 3FPBA (1.0 mM) at pH 7.4 in 100 mM phosphate buffer (*λ*_ex_ = 495 nm, *λ*_em_ = 565 nm).

To elucidate the mechanism, we studied the binding constant (*K*) between 3FPBA and NaSal, sugars and sugar alcohol using the fluorescence method with alizarin red S^14,24,25^ (Fig. 6). The compounds in decreasing order of *K* are Sor > Fru > NaSal > Glc (Table 1). The ordering of Sor > Fru > Glc corresponded with the case of PBA.^14^ Considering the similarities in the chemical structures of 3FPBA and PBA, and pH conditions (pH 7.4), the obtained *K* values are reasonable. Although the ordering of diols by the effect on *η*_0_ is not perfectly consistent with the ordering by *K*, the increasing effect of *η*_0_ almost reflected *K*. These results are indicative that the decrease in *η*_0_ in System A with the addition of 3FPBA and the increase in *η*_0_ in System B with the addition of sugar or sugar alcohol is associated with the formation of a competitive cyclic ester bond between 3FPBA and diol compounds.

**Table 1:** Binding constants (*K*) between 3FPBA and diol compounds at pH 7.4 in 100 mM phosphate buffer

Diol	Binding constant *K* (M^−1^)
ARS	1223
Sor	341
Fru	188
NaSal	166
Glc	1.65

**Fig. 7:**
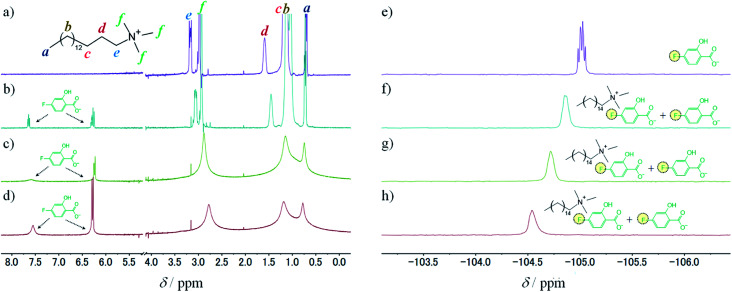
^1^H NMR spectra of 10 mM CTAB in 150 mM deuterium phosphate buffer (pD 7.8) (a) without FSal, (b) with 2.5 mM FSal, (c) with 5 mM FSal, and (d) with 10 mM FSal. ^19^F NMR spectra of 10 mM FSal in 150 mM phosphate buffer (pH 7.4) with 10% (v/v) D_2_O (e) without CTAB, (f) with 2.5 mM CTAB, (g) with 5 mM CTAB, and (h) with 10 mM CTAB.

To study the intermolecular interactions in the micellar systems, we performed ^1^H and ^19^F NMR spectroscopy. Because ^19^F NMR spectroscopy can reveal the hybridisation state of boron that binds to fluorobenzene, it is can be conducted to study the interaction between 3FPBA and diol compounds.^23,36,37^ Moreover, it is relatively easy to analyse signal changes in ^19^F NMR spectra because ^19^F NMR does not detect signals that are derived from the hydrocarbons of diol compounds, surfactants, and aromatic rings. For the NMR spectroscopy, we used FSal (a salicylic acid derivative with fluorine) instead of NaSal to investigate the interaction between FSal/3FPBA-complex and CTAB. To confirm the interaction between the quaternary ammonium ion of the cetyltrimethylammonium cation (CTA^+^) and the aromatic ring or carboxylate anion of FSal, we conducted ^1^H NMR spectroscopy of CTAB and CTAB with FSal (Fig. 7a–d).

The assignments of the chemical shift (*δ*) for the ^1^H signals of CTA^+^ are shown as symbols “*a*”–“*f*” (Fig. 7a). In the absence of FSal, *δ*_f_ (3.0 ppm) and *δ*_e_ (3.2 ppm) correspond to *δ* of the “*f*” and “*e*” symbols near the quaternary ammonium groups of CTA^+^, respectively (Fig. 7a). Both *δ*_f_ and *δ*_e_ shifted upfield with increasing FSal concentration to 2.8 ppm (Fig. 7b–d), which indicates an increase in the electron density around the quaternary ammonium groups of CTA^+^. One of the reasons for these upfield shifts is attributed to the interaction between the quaternary ammonium groups of CTA^+^ and the benzene ring of aromatic compounds.^38–40^ To verify this, we conducted ^19^F NMR spectroscopy of FSal. The FSal signal appeared at −105.0 ppm, and it shifted downfield with increasing CTAB concentration to −104.5 ppm (Fig. 7e–h), which indicates that the benzene ring of FSal interacts with the quaternary ammonium groups of CTA^+^, and the electron density near the aromatic ring decreases. Similar downfield shifts have been reported for fluorobenzene derivatives and cationic surfactant systems.^23,41,42^

**Fig. 8:**
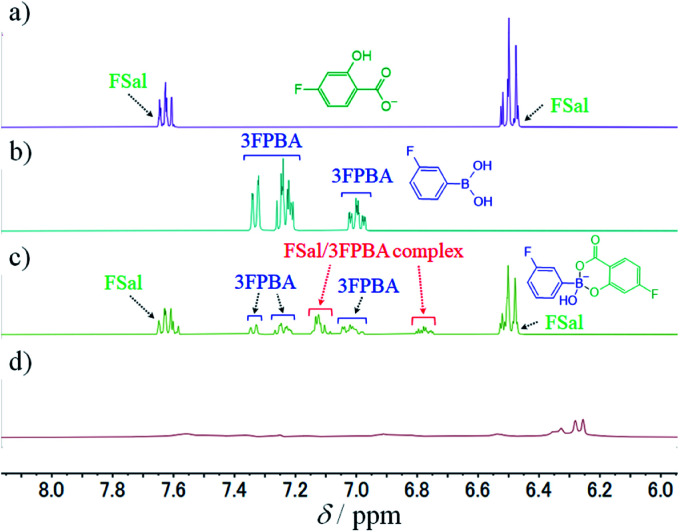
^1^H NMR spectra, using 150 mM deuterium phosphate buffer (pD 7.8), of (a) 10 mM FSal, (b) 10 mM 3FPBA, (c) 10 mM FSal with 5 mM 3FPBA, and (d) 10 mM FSal with 5 mM 3FPBA and 14 mM CTAB.

To study the interaction between FSal/3FPBA-complex and CTAB, we conducted ^1^H NMR spectroscopy, focusing on the signals in the range of 6–8 ppm that are derived from the benzene ring. The signals derived from FSal appeared at 6.5 and 7.6 ppm, whereas those from 3FPBA appeared at 7.0, 7.2, and 7.3 ppm (Fig. 8a and b). In the ^1^H NMR spectra of 10 mM FSal with 5 mM 3FPBA, new signals appeared at 6.8 and 7.1 ppm, which were attributed to FSal/3FPBA-complex (Fig. 8c). However, in the presence of 14 mM CTAB, the signals of the FSal, 3FPBA, and FSal/3FPBA-complex samples were too weak and complex to analyse, and new signals that appeared at approximately 6.3 ppm could not be assigned (Fig. 8d).

**Fig. 9:**
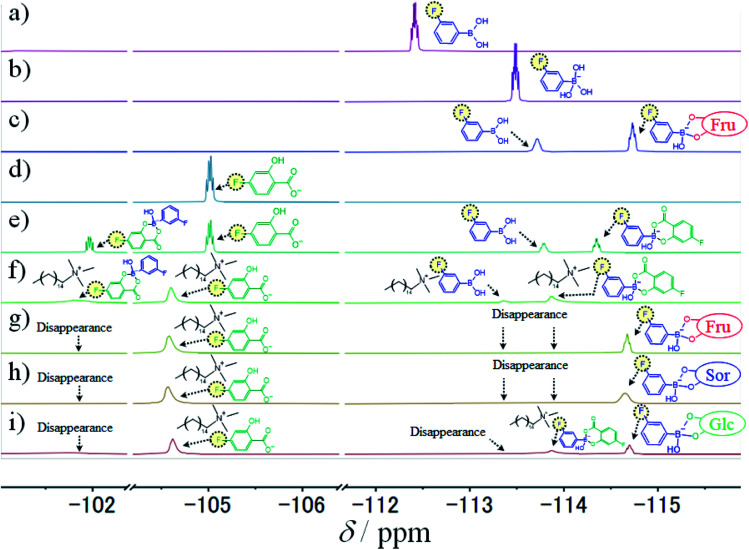
^19^F NMR spectra, using 10% (v/v) D_2_O and 150 mM phosphate buffer (pH 7.4 except in (a) and (b)), of (a) 10 mM 3FPBA (pH 5.0), (b) 10 mM 3FPBA (pH 11.0), (c) 10 mM 3FPBA with 10 mM Fru, (d) 10 mM FSal, (e) 10 mM FSal with 5 mM 3FPBA, (f) 10 mM FSal with 5 mM 3FPBA and 14 mM CTAB, (g) 10 mM FSal with 5 mM 3FPBA, 11.1 mM CTAB, and 0.64 M Fru, (h) 10 mM FSal with 5 mM 3FPBA, 11.1 mM CTAB, and 0.64 M Sor, and (i) 10 mM FSal with 5 mM 3FPBA, 11.1 mM CTAB, and 0.64 M Glc.

Because ^1^H NMR spectral results could not reveal the interaction between FSal/3FPBA-complex and CTAB, we performed ^19^F NMR spectroscopy. The signals of 3FPBA at pH 5.0 and 11.0 appeared at −112.4 and −113.5 ppm, respectively (Fig. 9a and b). Considering the p*K*_a_ of 3FPBA is 8.4,^23^ the signals at −112.4 and −113.5 ppm were ascribed to sp^2^- and sp^3^-hybridised boron, respectively. In the presence of 10 mM Fru with 10 mM 3FPBA at pH 7.4, the signal corresponding to sp^2^-hybridised boron was shifted to −113.7 ppm, and a new signal due to sp^3^-hybridised boron of 3FPBA/Fru-complex appeared at −114.7 ppm (Fig. 9c).^23^ Furthermore, the signal for 10 mM FSal appeared at −105.0 ppm (Fig. 9d). In the presence of 5 mM 3FPBA, four signals appeared at −102.0, −105.0, −113.7, and −114.4 ppm which correspond to FSal in FSal/3FPBA-complex, free FSal, free 3FPBA, and 3FPBA in FSal/3FPBA-complex, respectively (Fig. 9e). In the presence of 14 mM CTAB, the aforementioned four signals shifted downfield to −101.8, −104.6, −113.4, and −113.9 ppm, respectively (Fig. 9f), indicating that FSal/3FPBA-complex interacts with CTA^+^. To investigate the interaction between 3FPBA and sugar or sugar alcohol in the presence of FSal and CTAB, we performed ^19^F NMR spectroscopy. In the presence of Fru or Sor, the signals at −101.8, −113.4, and −113.9 ppm disappeared, the signal of free FSal remained at −104.6 ppm, and new signals derived from sp^3^-hybridised boron of 3FPBA/Fru or Sor-complex appeared at −114.7 ppm (Fig. 9g and h). Therefore, the amounts of the FSal/3FPBA-complex and free 3FPBA decreased, whereas those of the 3FPBA/Fru and Sor-complex increased with the addition of Fru or Sor to the CTAB/FSal/3FPBA micellar system. In addition, because the signals of the 3FPBA/Fru and Sor-complex samples did not shift as in the absence of CTAB, it indicates that 3FPBA/Fru and Sor-complex did not interact with CTA^+^ (Fig. 9c, g and h). In the presence of Glc, the signals at −101.8 and −113.4 ppm disappeared, the signal at −113.9 ppm slightly remained unchanged, the signal at −104.6 ppm did not shift, and the signal from sp^3^-hybridised boron of 3FPBA/Glc-complex appeared at −114.7 ppm (Fig. 9i). Similarly, these results showed that 3FPBA/Glc-complex did not interact with CTA^+^.

**Fig. 10:**
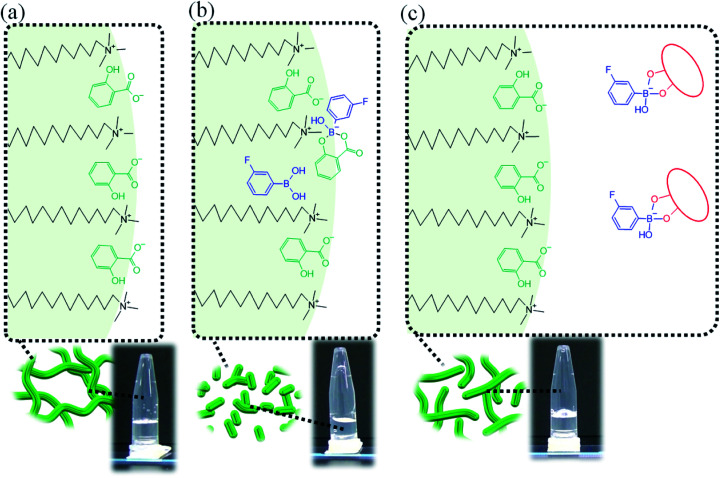
Proposed schematic diagram for the transformation of micelles in (a) System A, (b) System B and (c) System B with sugar or sugar alcohol.

We propose the following mechanism of the viscosity change in this system. In System A, CTA^+^ interacts with the salicylate anion (Sal^−^),^1,2^ as shown in Fig. 7, which weakens the electrostatic repulsion with the head groups of CTA^+^. This tightens the packing of CTA^+^, leading to the formation of adequately long WLMs (Fig. 10a).

Previously, it has been reported that on adding diol compounds such as sugars into the CTAB/3FPBA micellar system, 3FPBA forms cyclic ester bonds with diol compounds, leading to an increase in the sp^3^ hybridised boron of 3FPBA, which does not interact with CTA^+^.^23^ In this study, 3FPBA forms a cyclic ester bond with Sal^−^ (Fig. 8c and 9e). Although we expected that FSal/3FPBA-complex would not interact with CTA^+^, they interacted with each other (Fig. 9f). Based on these interactions, we assumed that the influence of FSal/3FPBA-complex on CTA^+^ is different from that of free Sal^−^ or free 3FPBA. This presumption supports the occurrence of phase separation in System E, in which the content of NaSal/3FPBA-complex is higher than that in System B (Fig. 2). Owing to the strengthening of the interaction between NaSal/3FPBA-complex and CTA^+^, the packing state of CTA^+^ changed (Fig. 10b), which shortened the WLMs and decreased the viscosity of System B. However, upon the addition of sugar or sugar alcohol to System B, 3FPBA bound to Sal^−^ partially binds with sugar or sugar alcohol (Fig. 9g–i), leading to a decrease in the amount of NaSal/3FPBA-complex interacting with CTA^+^, and an increase in the amount of 3FPBA/Fru or Sor-complex which does not interact with CTA^+^ (Fig. 10c). The changes in the molecular interactions induce the elongation of the WLMs and increase the viscosity of System B with sugar or sugar alcohol. Because Fru and Sor tends to bind with 3FPBA unlike Glc (Fig. 9g–i), the increase in viscosity of System B is larger for Fru and Sor than that for Glc (Fig. 3c). However, we cannot explain the difference in the increase in viscosity between Fru and Sor in System B.

In summary, the viscosity changes of the system are caused by the transformation of WLMs associated with the formation of competitive cyclic ester bonds between 3FPBA and diol compounds.

## Conclusions

In conclusion, we prepared a novel micellar system utilising a PBA derivative whose viscosity increased in response to diol compounds such as sugar and sugar alcohol. This unique concept of utilising competitive cyclic ester bonds between PBA derivatives and diol compounds provides a new possibility for stimuli responsive WLMs as smart materials.

## Conflicts of interest

There are no conflicts to declare.

## Supplementary Material
